# The Effect of Particle Size on the Cytotoxicity of Amorphous Silicon Dioxide: An in Vitro Toxicological Study

**DOI:** 10.31557/APJCP.2021.22.2.325

**Published:** 2021-02

**Authors:** Athena Rafieepour, Mansour R. Azari, Jalal Pourahmad Jaktaji, Fariba Khodagholi, Habibollah Peirovi, Yadollah Mehrabi, Yousef Mohammadian

**Affiliations:** 1 *Student Research Committee, School of Public Health and Safety, Shahid Beheshti University of Medical Sciences, Tehran, Iran. *; 2 *School of Public Health and Safety, Shahid Beheshti University of Medical Sciences, Tehran, Iran. *; 3 *School of Pharmacy, Shahid Beheshti University of Medical Sciences, Tehran, Iran. *; 4 *Neuroscience Research Center, Shahid Beheshti University of Medical Sciences, Tehran, Iran. *; 5 *Nanomedicine and Tissue Engineering Research Center, Shahid Beheshti University of Medical Sciences, Tehran, Iran. *; 6 *Department of Occupational Health Engineering, Tabriz University of Medical Sciences, Tabriz, Iran. *

**Keywords:** Amorphous silicon dioxide, nanoparticles, microparticles, A549 cell lines, toxicity, in vitro

## Abstract

**Introduction:**

Amorphous silicon dioxide (A-SiO_2_) is abundant in the Earth’s crust, the A-SiO_2_ nano and microparticles are released into the air through industrial and manufacturing activities. Due to the limited available toxicological information, the objective of the present study was to evaluate the toxicity of different sizes of A-SiO_2_ particles on the A549 cell-lines in an in vitro study.

**Materials and Methods:**

The A-SiO_2_ particles in two categories of nano (10-100 nm) and micro (< 5um) were used in this study. The human lung A549 cell-line was exposed to either nano- or micro-sized A-SiO_2_ particles at 10, 50, 100, and 250 μg/ml, and the effects were investigated.

**Results:**

The cytotoxicity of A-SiO_2_ nano and microparticles in both 24- and 72-hour exposure times resulted in decreased cell survival, mitochondrial membrane potential, and increased ROS generation which was concentration-time dependent (P <0.05) but glutathione content was not affected in a time-dependent manner. Cytotoxicity of nanoparticles, contrary to the previous study, was not higher than microparticles in the comparable dose and exposure times.

**Conclusion:**

The rate of ROS generation in the A549 cell-line exposed to A-SiO_2_ nanoparticles was higher than microparticles. And at the same time, cell survival for exposed cells to A-SiO_2_ nano and microparticles were higher for nanoparticles in shorter exposure periods and was inversely concentration- and time-dependent. Further studies on exploring the effect of size and its possible toxic mechanism are recommended to achieve a more credible risk assessment.

## Introduction

Airborne contaminants are one of the important causes of morbidity in the world, and according to a WHO (2014) report, there are 7 million premature deaths per year as a result of exposure to them. Airborne particle pollutants are divided into three groups of large particles (PM10), fine particles (PM2.5) and ultrafine particles (UFP, PM0.1), and the most harmful effects can be observed in ultrafine particles with an aerodynamic diameter of less than 100 nm (Breitner et al., 2011; Franck et al., 2011; Luch, 2012). An increase in the surface-to-volume ratio, as well as an increase in the number of very small particles, is one of the reasons that can increase these toxic effects (Sioutas et al., 2005) and since respiratory tract and lungs with an entry rate of 200 billion particles per day are directly in contact with pollutants, impairing the function of the respiratory system is very prevalent throughout the world (Donaldson et al., 2005; Cohen et al., 2017). Also, particles in fine and nanometer sizes can easily pass through the membrane and damage vital organs systemically (Hillyer and Albrecht, 2001; Donaldson et al., 2004; Fischer and Chan, 2007), although the mechanism of this effect is not clear, and some researchers believe that nanoparticles do not differ significantly in their toxic effects compared to microparticles (Karlsson et al., 2009). However, the production of nanoparticles and their widespread use in modern science, in comparison with microparticles, has increased the human exposure to these materials both intentionally and accidentally (Peixe et al., 2015).

The Organization for Economic Cooperation and Development (OECD) has reported that the use of common nanoparticles such as silicon dioxide nanoparticles are expected to increase in the future (Leung et al., 2012). Amorphous silicon dioxide particles (A-SiO_2_) are widely used in various processes, such as drug delivery and biomedicine (Sergent et al., 2012). These nanoparticles are unique in their physicochemical properties and are a byproduct in the manufacture of several types of commercial products. Also, harvesting agricultural products or burning waste in furnaces could expose the general population to A-SiO_2_ (Merget et al., 2002; Barik et al., 2008; Napierska et al., 2010; Wang et al., 2015). Besides, respirable silicon dioxide particles are one of the most common occupational pollutants throughout the world (Dahmann et al., 2008) that can expose workers and people living around plants, mines, or dry areas (Greenberg et al., 2007; Santos et al., 2010). Generally, accurate toxicological evaluation has been recommended by many health experts (Karlsson et al., 2009).

The International Agency for Research on Cancer (IARC) has identified A-SiO_2_ as non-carcinogenic to humans stating that it has less toxic effects compared to crystalline forms (Raju and Rom, 1998). In some studies, inhalation exposure to A-SiO_2_ nanoparticles in animal subjects has been partially associated with reversible inflammation in the respiratory system and changes in blood parameters, but these studies do not provide evidence of the progression of fibrosis in the lungs (Shin et al., 2017). However, in an epidemiological study, the occurrence of four cases of silicosis was reported among people exposed to pure A-SiO2 (Merget et al., 2002). In some cohort studies, A-SiO2 has been known to increase mortality due to pulmonary diseases (Puntoni et al., 1988; Checkoway et al., 1993), and the results of some studies indicated that inhalation of A-SiO_2_ nanoparticles could result in significant pulmonary toxicity in comparison to its crystalline form (Kaewamatawong et al., 2005; Yazdi et al., 2010). The importance of the study for comparison the toxicity of silicon dioxide micro and nanoparticles has been expressed by some researchers (Sahu et al., 2016). On the other hand, most of the particles’ toxicity information was shown in animal models (Szalay, 2012a) that raises concerns about generalizing the toxicity effects in humans (May et al., 2009).

Laboratory work focused on investigating the effect of A-SiO_2_ particles could play an important role in evaluating the adverse toxicity risk to humans; hence the objective of the present study was to investigate the toxicological effects of A-SiO_2_ particle sizes of micron and nanometer in the A549 human lung cell line.

## Materials and Methods

*Materials and reagents*

A-SiO_2_ nanoparticles in the diameters of about 15-20 nanometers were purchased from the US Research Nanomaterials company. A-SiO_2_ of micron size was not available in Iran. Therefore, with the consultation of the Faculty of Geology at the Shahid Beheshti University, Yellow Namibian opal stone was purchased and ground by a ball mill (Siebtechnik, Holland (380 V, 0.75 KW) for 40 hours. The powdered material was treated to the extraction of impurities such as metals from ball milling, according to the method recommended by Zong et al., (2015), using hydrochloric acid in 2M concentration and concentrated nitric acid. 

*Physicochemical properties of A-SiO*_2_ *particles*

The chemical structure and purity of the ground A-SiO_2_ microparticles were confirmed using the XRD diffraction (STOE-STADV, Germany) and the ICP-OES (Agilent 7700, USA), respectively. The size of A-SiO_2_ microparticles was measured by the phase-contrast microscopy (PCM, Dialux 22EB- Germany) method recommended by Stefaniak et al., (2007). The A-SiO_2_ nanoparticles’ sizes were also confirmed using a transmission electron microscope (TEM, Philips CM30, Netherlands). 

The particle size distributions were measured in a 100 μg/ml concentration of A-SiO_2_ nano and microparticles in culture media. The suspension was stored for 25 minutes at 25°C for 1 hour and then dispersed with a sonicator for 20 minutes at 240V and 60 Hz frequency. Particle size distribution was measured using a DLS (Nanophox 90-246V, Germany). The surface area of A-SiO_2_ nano and microparticles were analyzed by Brunauer-Emmett-Teller (BET, Tristar II-3020, US). 

*Cell culture and exposure to A-SiO*_2_ *particles*

The A549 cell line was purchased from the Pasteur Institute in Iran and cultured in a Dulbecco’s Modified Eagle Medium (DMEM, Bio Idea Co., Iran) + 10% Fetal Bovine Serum (FBS, GIBCO, USA) and 1% Penstrep (GIBCO, USA) in an incubator at 37°C and 5% CO_2_. After reaching to an 80% confluency of cells, the supernatant of each plate was replaced by serum-free DMEM and DMEM containing A-SiO_2_ particles for control and exposure groups, respectively. Toxicological tests were performed at four concentrations of 10, 50, 100, and 250 μg/ml of A-SiO_2_ nano and microparticles with 24 and 72 hours exposure times according to the previous studies that showed that the entrance of SiO_2_ particles into the perinuclear of A549 cells occurred in ≥ 24 h exposure time (Ahamed, 2013; Nowak et al., 2014). Serum-free medium was used to prepare different concentrations of A-SiO_2_ nano and microparticles and homogenized for 20 minutes before use in a sonicator device. 

*Cell survival assay*

1×10^4^ cells per well were seeded in a 96-well plate and were exposed overnight to nano and microparticles of A-SiO_2_. After 24 and 72 hours exposure periods, MTT reagent (3- (4.5-dimethylthiazol- 2yl)-2.5-diphenyltetrazolium bromide, Sigma) was added to each well and the color change resulting from the conversion of MTT salt to Formazan as a result of the mitochondrial activity of succinate dehydrogenase in survival cells (Mosmann, 1983) was measured by an ELISA plate reader (Biotek ELx 800, USA at 570 nm). Results were calculated by the ratio of the average cell survival in the exposed group to the average cell survival in the control group ×100.

*Mitochondrial Membrane Potential assay*

1×10^4^ cells per well of a 96 black well plate were exposed to different concentrations of nano and microparticles of A-SiO_2_ at 24 and 72 hours exposure times. 200 µl of DMEM containing Rhodamine 123 at a concentration of 2 μM was added to each well for 60 minutes before the test (Johnson et al., 1980). The fluorescence intensity of Rhodamine 123 was measured by a Multi-Mode Reader (Synergy-HTX, BioTEk, USA) at 485 and 528 nm of excitation and emission wavelengths, respectively. Finally, the mitochondrial membrane potential was calculated based on the difference in fluorescence intensity between the control and test groups (Villena et al., 2013; Sakamuru et al., 2016) and reported in comparison to the control group. 

*ROS assay*

1×10^4^ cells per well in a 96 black well plate were exposed to different concentrations of A-SiO_2_ nano and microparticles at 24- and 72-hours exposure periods. The intensity of fluorescence induced by adding DCFH-DA reagent and converting to DCF was measured by a Multi-Mode Reader (Synergy-HTX, BioTEk, USA) at 485 and 525 nm of excitation and emission wavelengths, respectively and was considered intracellular ROS index (LeBel et al., 1992). Results were calculated by the ratio of the average ROS generation in the exposed group to the control group ×100.

*Intracellular glutathione content assay*

The A549 cellular glutathione content in 24 and 72 hours exposure periods to A-SiO2 micro and nanoparticles was measured according to the method recommended by Ellman (1959). In this test, the lysis cells were exposed to a DTNB reagent (5,5’-Dithiobis [2-nitrobenzoic acid]) at 2 mg/ml, and the yellow color resulting from the DTNB depletion by free protein thiols to TNB was measured by an ELISA reader (Biotek ELx 800, USA) at a wavelength of 405 nm (Requejo et al., 2010). The GSH content in exposed cells was reported in comparison to GSH content in control cells. 

*Statistical analysis*

Toxicity tests were repeated three times and the results demonstrated as mean ± standard deviation. SPSS software version 16 and t-test independent sample and one-way ANOVA tests were used to analyze the data obtained from toxicology tests and results showed that in a 95% confidence interval. 

## Results

*Physicochemical properties of A-SiO2 nano and microparticles*

**Figure 1:**
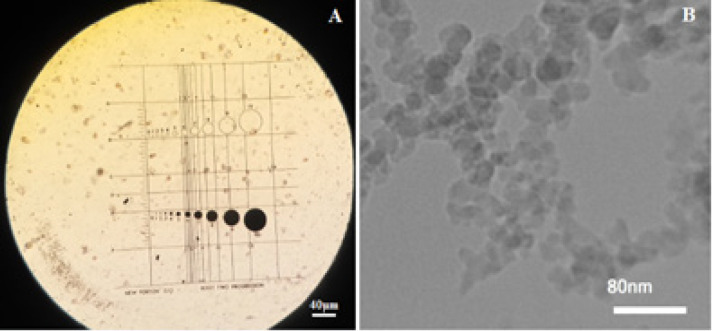
A-SiO_2_ A) microparticles (PCM) and B) nanoparticles (TEM)

According to the information provided by US Research Nanomaterials corporation, the specific surface area and purity of A-SiO_2_ nanoparticles were 170 - 200 m^2^/g and 99.5%, respectively (US-Research-Nanomaterials-Inc, 2020). The physical properties of A-SiO_2_ microparticles were analyzed by BET and ICP-OES (Table 1). The results showed that the specific surface area and purity of A-SiO_2_ microparticles were 34.71 ± 0.5 m^2^/g and 99.75%, respectively. The size of A-SiO_2_ nano and microparticles in their powdery state (Figure 1) and in suspension in DMEM are shown in Table 2.

**Figure 2:**
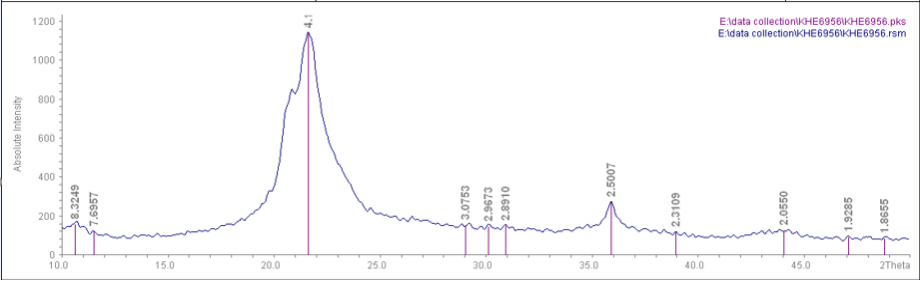
XRD Analysis for A-SiO_2_ Microparticles

After the purification process, the amorphous nature of SiO2 microparticles was confirmed by X-Ray diffraction, and did not show a peak in the 2θ = 27-26º (Wang et al., 2012) (Figure 2).

*Cell survival rate*

A549 cell-line exposure to A-SiO_2_ nano and microparticles in all concentrations and two time periods showed concentration and time-dependent reduction in cell survival. Reduction in cell survival during 24-hour exposure time with A-SiO_2_ nano and microparticles expect 10 μg/ml concentration of A-SiO_2_ nanoparticles compared to the control group was statistically significant (P <0.05). A549 cell-line exposed for 72 hours to A-SiO_2_ nano and microparticles demonstrated significantly lower cell survival than the control group (P <0.05).

The results of this study showed a significant difference in A549 cell survival when exposed to A-SiO2 microparticles compared to nanoparticles in all concentrations (P <0.05). It must be added that cell survival at 24-hour exposure time with all concentrations of A-SiO_2_ except for the 250 μg/ml microparticles was lower than nanoparticles, and at 72-hour exposure time at all concentrations of A-SiO_2_ nanoparticles except 10 μg/ml was lower than A-SiO_2_ microparticles.

*A549 mitochondrial membrane potential rate*

The decrease in the mitochondrial membrane potential of exposed cells in all concentrations of A-SiO2 nanoparticles, except 10 μg/ml, in 72-hour exposure, was significantly higher than the control group and demonstrated a decrease proportional to exposure time and an increase in concentration (P <0.05). Also, the decrease in the A549 mitochondrial membrane potential in cells exposed to all concentrations of A-SiO_2_ microparticles was higher compared to exposure to A-SiO_2_ nanoparticles in both exposure periods (P <0.05).

*Intracellular ROS rate*

The decrease in the mitochondrial membrane potential of exposed cells in all concentrations of A-SiO_2 _nanoparticles, except 10 μg/ml, in 72-hour exposure, was significantly higher than the control group and demonstrated a decrease proportional to exposure time and an increase in concentration (P <0.05). Also, the decrease in the A549 mitochondrial membrane potential in cells exposed to all concentrations of A-SiO_2_ microparticles was higher compared to exposure to A-SiO_2_ nanoparticles in both exposure periods (P <0.05).

*Intracellular glutathione content rate*

In the 24-hour exposure to A-SiO_2_ microparticles, the intracellular glutathione content was higher than the control group but in the 72-hour exposure to A-SiO_2_ microparticles, the intracellular glutathione was significantly lower than the control group and showed concentration-dependent behavior.

The intracellular glutathione content in the 24-hour exposure to all concentrations of A-SiO_2_ nanoparticles except 250 µg/ml, was lower than the control group. In the 72-hour exposure to A-SiO_2_ nanoparticles, the amount of glutathione in all concentrations was higher than the control group and showed a negative proportionality to higher concentrations. Also, following the increase of exposure time to A-SiO_2_ microparticles, the decrease in intracellular glutathione content was more than A-SiO_2_ nanoparticles, and increasing the exposure time resulted in decreased intracellular glutathione content.

**Table 1:** Purification Efficiency of A-SiO_2_ in Micron Sizes by ICP-OES

Metal impurity as (ppm)	Ag	Al	Be	Cd	Cr	Cu	Fe	Li	Mn	Ni	Pb
Before extraction	0.5>	1148.1	1.46	0.11	38.37	15.51	8077.24	20.78	43.31	14.26	11.16
After extraction	0.5>	451.15	0.39	0.1>	31.12	3.76	2055.41	20.45	5.9	4.08	4.34

**Table 2:** Powdery Size, DLS (in DMEM, 50 µg/ml Concentration, 25^o^C) and Zeta Potential (in Analar Water, 25^o^C) Analysis Results for Nano and Micro A-SiO_2_ Particles

	Average size in powdery state (nm)	Average size in suspension state (nm)	Zeta potential (mV)
A-SiO_2_ microparticles	3670	9927.08	-32.2
A-SiO_2_ nanoparticles	≤ 20	220. 58	-34.2

**Figure 3:**
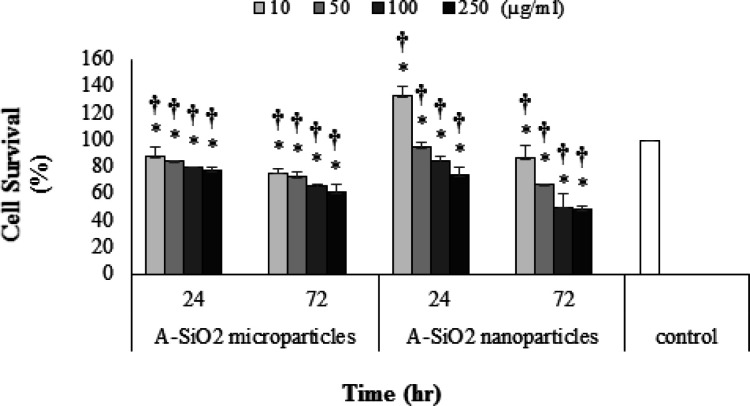
A549 Cell-Line Survival in 24- and 72-hour Exposure Periods and 10, 50, 100 and 250 µg/ml Concentrations of A-SiO_2_ Nano and Microparticles. (Results of mean ± SD of cell survival percent from three experimental tests).* † Significant statistical differences; 1) between experimental groups compared to control and 2) between comparative experimental groups exposed to micro and nano-sized particles (p-value <0.05)

**Figure 4:**
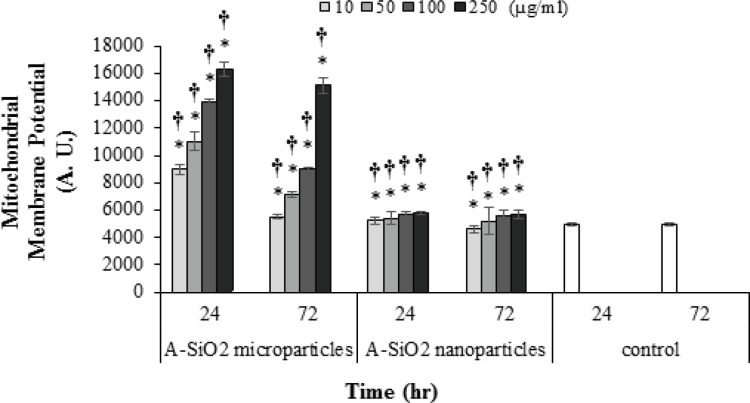
A549 Cell-Line Mitochondrial Membrane Potential Reduction in 24- and 72-hour Exposure Periods and 10, 50, 100 and 250 µg/ml Concentration of A-SiO_2_ nano and Microparticles. (Results of mean ± SD of absorbance rate from three experimental tests). * † Significant statistical differences; 1) between experimental groups compared to control and 2) between comparative experimental groups exposed to micro- and nano-sized particles (p-value <0.05)

**Figure 5:**
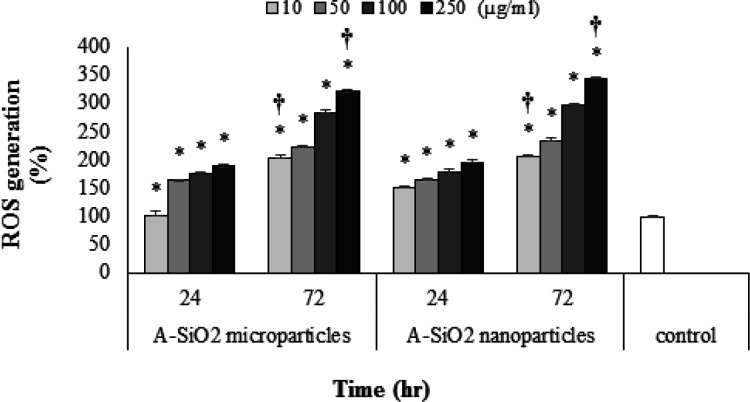
Intracellular ROS Generation in 24- and 72-Hour Exposure Periods to 10, 50, 100 and 250 µg/ml Concentrations of A-SiO_2_ Nano- and Microparticles. (Results of mean ± SD of ROS generation percent from three experimental tests). * † Significant statistical differences; 1) between experimental groups compared to control and 2) between comparative experimental groups exposed to micro- and nano-sized particles (p-value <0.05)

**Figure 6:**
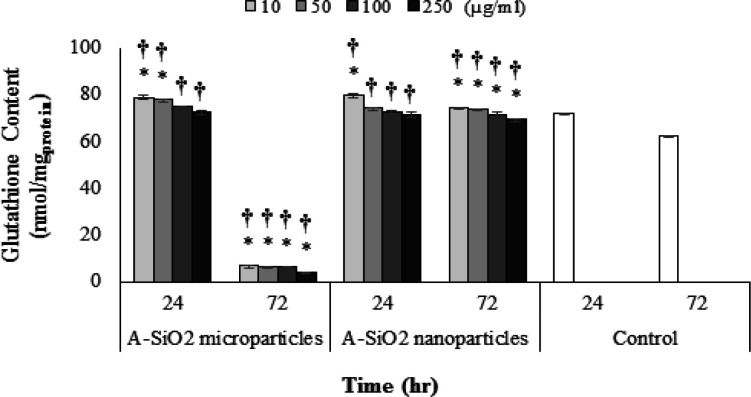
Intracellular Glutathione Content in 24- and 72-Hour Exposure time to 10, 50, 100 and 250 µg/ml Concentrations of A-SiO_2_ Nano and Microparticles. (Results of mean ± SD of GSH concentration per mg of cell's protein from three experimental tests). * † Significant statistical differences; 1) between experimental groups compared to control and 2) between comparative experimental groups exposed to micro- and nano-sized particles (p-value <0.05)

## Discussion

Silicon dioxide particles are among the most commonly used in industries, constructions, and cities (Hurley et al., 2003; Karlsson et al., 2008; Faraji and Wipf, 2009; Rivas-Sánchez et al., 2009; Mishra, 2015). The size of silicon dioxide particles and the toxicity of nanoparticles has been a health concern in spite of their benefits in industries and pharmaceutical applications (Szalay, 2012b). Since most of the toxicological information of nanoparticles has been derived from experiments on the animal species (Szalay, 2012b), the present study was designed to investigate the effect of SiO_2_ particles on the human pulmonary cell-line.

The findings of this study showed that the concentration of both nano and microparticles of A-SiO_2_, and their exposure time affected the toxicity of the exposed A549 cell-line. Generally, this study demonstrated a lower cell survival, mitochondrial membrane potential, and higher ROS generation in cells exposed to A-SiO_2_ microparticles compared to nanoparticles. Decrease of the intracellular glutathione content, A549 mitochondrial membrane potential, and cell survival and the increased ROS generation of exposed cells were concentration- and time-dependent. In a study by Lin et al., (2006) the A-SiO_2_ particle toxicity in human bronchial cells was investigated in two sizes of 15 and 46 nm, and the results showed that the toxicities of the two nanoparticles were similar to each other. These results seemed fairly consistent with the present findings in terms of cell survival compared to the control group concluding that the toxic effect of A-SiO_2_ was through damage to the mitochondrial membrane. However, in contrast to the present study, Napierska et al., (2009) stated that the cell viability of the EAHY926 cell-line exposed to A-SiO_2_ nanoparticles was lower than A-SiO_2_ microparticles.

Contrary to the results of the present study, He et al., (2009) stated that the toxicity of A-SiO_2_ nanoparticles was higher than A-SiO_2_ microparticles in the MDA-MB-468 and COS-7 cells. They reported that nanoparticles had a higher potential of penetration through the lysosomes compared to microparticles and hence have a more lethal effect on the cells through the rupturing of the lysosome and cell lysis. The disparity of the present findings with those of He and et al. could be explained through differences in test concentrations and the nature of the cell lines used in the two studies. However, Chen et al., (2004) also confirmed the results of the present study by examining the effect of A-SiO_2_ nano and microparticles on the Wistar rat lung, and they reported a higher incident rate of lung fibrosis for microparticles, justifying the lower toxicity of A-SiO_2_ nanoparticles through the easier movement in and out of the exposed cells, and pointed out that this could be a key factor in reduced toxicity. Guichard et al.,’s findings (2012) were in line with the results of the present study and stated that the toxicity of the cells exposed to particles is dependent on their concentration and exposure time as well as their size.

Despite findings of our study, which demonstrated lower toxicity for the A-SiO_2_ nanoparticles compared with A-SiO_2_ microparticles for a short exposure period of 24 hours, the cells exposed to nanoparticles had a higher ROS generation, as well as an increased the production rate of glutathione content which could nullify the potent ROS. The result of the present study defies other authors who have reported the amount of intracellular ROS as a reduction factor in the content of intracellular glutathione (Chen et al., 2018).

In conclusion, the results of the present study demonstrated the toxicity of A549 human cell lines exposed to A-SiO2 nano and microparticles were concentration and time dependent. In shorter exposure periods, nanoparticles had lower toxicity than microparticles in similar exposure concentrations. Generally, it was demonstrated that particle size is not the only factor affecting the toxicity, and concentration and exposure time are also important factors. The authors of this study recommend further studies exploring the possible mechanisms of toxicity phenomena observed in this study.
